# Gender-specific trends of educational inequality in diagnosed diabetes from 1999 to 2014 in Hong Kong: a serial cross-sectional study of 97,481 community-dwelling Chinese adults

**DOI:** 10.1186/s12963-021-00268-x

**Published:** 2021-10-10

**Authors:** Gary Ka-Ki Chung, Francisco Tsz Tsun Lai, Eng-Kiong Yeoh, Roger Yat-Nork Chung

**Affiliations:** 1 The Jockey Club School of Public Health and Primary Care, Faculty of Medicine, The Chinese University of Hong Kong, Shatin, New Territories, Hong Kong China; 2 CUHK Institute of Health Equity, The Chinese University of Hong Kong, Hong Kong, China; 3 Department of Pharmacology and Pharmacy, The University of Hong Kong, Hong Kong, China

**Keywords:** Community trends, Diabetes, Education, Gender, Income, Inequalities

## Abstract

**Background:**

Gender differences in the trend of educational inequality in diabetes have been widely observed in the Western populations, indicating the increasing importance of educational attainment as a social determinant of diabetes among women. Nonetheless, relevant evidence is scarce in developed Asian settings for comparisons. This study examined the gender-specific trends of educational inequality in diagnosed diabetes in Hong Kong between 1999 and 2014.

**Methods:**

A series of eight territory-wide population-representative samples of 97,481 community-dwelling Hong Kong Chinese adults aged 45 or above were surveyed between 1999 and 2014. Regression-based Relative Index of Inequality (RII) and age-standardized Slope Index of Inequality (SII) were adopted to examine the extent and trend of gender-specific educational inequality in self-reported physician-diagnosed diabetes.

**Results:**

Age-standardized prevalence of diabetes increased in both genders over time, with a steeper surge among men. In addition, educational inequalities in diabetes, in both relative and absolute terms, significantly widened among women over the study period (annual RII change = 1.04; 95% CI = 1.02–1.07, annual SII change = 0.36%; 95% CI = 0.16–0.56%), with the peak in 2011 (RII = 2.44; 95% CI = 1.83–3.24, SII = 9.21%; 95% CI = 6.47–11.96%). However, no significant widening inequality was found among men. Further adjustment for household income level did not attenuate the observed educational inequality**.**

**Conclusions:**

Despite a greater increase in diabetes prevalence among men, disparity in diabetes substantially widened across education levels among women in the past decade in Hong Kong. The gender perspective should be taken into considerations for policy making to alleviate the prevalence surge and rising educational inequality in diabetes.

**Supplementary Information:**

The online version contains supplementary material available at 10.1186/s12963-021-00268-x.

## Background

Since the 1990s, the global prevalence of diabetes has doubled [1], reaching 383 million in 2016 [2]. Despite being among the top killers, diabetes is also a strong risk factor of many other global leading causes of death, such as cardiovascular diseases and cancers [3, 4]. In addition, diabetes is associated with a range of complications such as blindness, kidney failures and amputations [5], thereby resulting in a high disability burden which requires intensive long-term management [6]. Despite global attention on diabetes control, a further surge in prevalence of diabetes to almost 600 million is expected by 2035 [7].

In addition, diabetes is also a highly socioeconomically patterned disease, in favor of the advantaged groups, especially in the developed world regions [8, 9]. According to a systematic review on socioeconomic positions and incidence of diabetes, low education is most consistently associated with an increased risk of diabetes when compared to other socioeconomic indicators [9]. The authors argued that the knowledge and skills acquired through education determine the responsiveness to health information [9], since the more educated people tend to have more resources and be able to filter and follow the information efficiently to their own benefits [10]. As previous research suggested the role of the emergence of digital media, as well as online health information-seeking practices, over the recent decades in exacerbating social inequalities in health [11], disparity in diabetes across educational levels is expected to have widened.

Previous studies in the Western populations generally showed a widening educational inequality in diabetes over time [12, 13]; nonetheless, the widening inequality differs by genders. Consistently found in studies in Spain, England and Canada, educational inequality in diabetes widened more apparently among women, with a greater increase in prevalence among the less educated women [14–16]. However, evidence in Asian settings is scarce. While recent cross-sectional studies in Hong Kong and China supported gender-specific associations of education with both prevalent and incident diabetes, it did not assess their temporal trends [17, 18]. To the best of our knowledge, we found two relevant Asian studies conducted in South Korea, one of which reported persistent educational inequality in diabetes among women but not among men between 2007 and 2014 [19], whereas another one showed some signs of improvement in the educational inequality in diabetes among women after 2010 [20]. Taken together, trends of educational inequality in diabetes appear to be gender-specific; however, whether the pattern can generally be applied to different Asian populations has yet to be confirmed.

The present study examined the gender-specific trends of educational inequality in diabetes from 1999 to 2014 in Hong Kong, a highly developed Asian setting. Understanding the trends in Hong Kong is particularly relevant to the rapidly developing China and other emerging economies in Asia, since Hong Kong had experienced rapid economic and epidemiologic transitions in the second half of the twentieth century [21] and has also become a leading digital economy in Asia [22]. The experience in Hong Kong could serve as an exemplar to inform resource allocation and prevention strategies for reducing diabetes and the associated cardiovascular disease burden in these emerging Asian economies.

## Methods

### Data collection and study population

The present study adopted a serial cross-sectional design using eight territory-wide population-representative household surveys, the Thematic Household Survey (THS) commissioned by the Census and Statistics Department (C&SD) of the Hong Kong Government in 1999, 2001, 2002, 2005, 2008, 2009, 2011 and 2014 [23]. The target population of THS includes all land-based non-institutional Hong Kong Resident Population [24]. At least 95% of the Hong Kong Resident Population were covered in these surveys at their corresponding periods, with response rates between 75 and 79%. In the present study, the final sample included 97,481 adults aged 45 years or above.

### Dependent variable

In the surveys conducted in 1999, 2001, 2002 and 2005, respondents first responded to the question “do you have any diseases that require long-term follow-up?”, and, if so, were then asked whether they had diabetes. The corresponding question in surveys conducted in 2008, 2009, 2011 and 2014 was slightly amended to “have you ever been told by a western medicine practitioner that you had the following chronic health conditions?”, with diabetes as a response option. Self-reported status of diabetes was adopted as the binary dependent variable in all surveys.

### Independent variables

Sociodemographic indicators, in terms of gender, age (categorized into five groups from “45–49 years” to “65 years or above”), marital status (categorized into “non-married” for those never married, divorced, separated or widowed, and “married” including cohabitation), and household size (categorized into five groups from “single household” to “five members or above”), were obtained for standardization or confounding adjustments. Socioeconomic characteristics including educational attainment and monthly household income level were also collected in the present study. The highest attained education levels of respondents were obtained and categorized into “Tertiary level,” “Secondary level,” “Primary level” and “Below primary level.” Self-reported information on monthly household income levels, in Hong Kong dollars (7.8 HKD = 1 USD), was also collected with 12 ordinal income groups from “below $4000” to “$50,000 or above.” Specifically, for descriptive analyses, monthly household income levels were re-categorized into “$9999 or below,” “$10,000–24,999,” “$25,000–49,999” and “$50,000 or above.”

### Statistical methods

Descriptive characteristics of respondents across survey years, stratified by genders, were presented as count data with column percentages. When comparing the prevalence of diabetes across education and household income levels over years, age standardization was adopted based on the overall and gender-specific age structures of the mid-2016 Hong Kong population [25]. If no respondents were identified in an age-specific education or income group, the respective age-standardized prevalence could not be calculated and thus would not be provided.

#### Multivariable analyses

##### Relative Index of Inequality and age-standardized Slope Index of Inequality

To quantify the extent of relative and absolute educational inequality in diabetes, two major summary measures, the Relative Index of Inequality (RII) and the age-standardized Slope Index of Inequality (SII), were employed [26]. Fractional rank scores, scaled from 0 (highest education/household income level) to 1 (lowest education/household income level), were calculated for all the education and household income categories in each year to represent the proportion of respondents with a socioeconomic status higher than a given individual plus half of the proportion of the individual’s own category. To account for the variations of socioeconomic distributions by genders, gender-specific fractional rank scores were also derived. The resulted fractional rank scores were then adopted as the independent variables in generalized linear regression models for binomial dependent variables [26]. When applying the logarithmic link function for relative measures, RIIs could be assessed by the coefficients of fractional rank scores in the models, whereas when using the identity link function for absolute measures, age-standardized SIIs could be estimated by the age-specific coefficients of fractional rank scores. Age standardization for SIIs allows a meaningful comparison of the extent of absolute inequality across populations in different survey years due to the changing age structure over the study period. Adjustments for age-groups, gender (not adjusted in gender-specific models), marital status and household size were applied to the regression models. Regarding the interpretations, RIIs above 1 and SIIs above 0 represent inequality with higher odds of having diabetes among the less educated or income-poor groups in relative and absolute terms, respectively. For instance, if RII and SII are estimated to be 1.5 and 10%, respectively, the hypothetically least educated group (fractional rank score = 1) will have 50% higher odds of having diabetes and be 10% more prevalent when compared to the hypothetically most educated group (fractional rank score = 0). The model-robust approaches were employed to obtain standard errors and to construct 95% confidence intervals (CI) and *p* values [26].

##### Trend of RIIs and SIIs over the study period

Interaction terms were additionally included in the generalized linear models in order to assess the trends of relative and absolute educational inequalities over time. A two-way interaction term (fractional rank scores of education * survey years) was used for the trend of RIIs, while a three-way interaction term (factional rank scores of education * survey years * age-groups) was adopted for the trend of age-standardized SIIs [26]. Changes in educational inequality over time could be represented by the coefficients of the corresponding interaction terms. An additional model was also employed to examine whether household income could explain the trend of educational inequality by putting fractional rank scores of education and household income, and their associated interaction terms into the same regression model. Model-robust approaches were employed to test for significance of the trends.

All data analyses were conducted using statistical software R 3.4.0, and *p* values < 0.05 were regarded to be statistically significant.

## Results

### Descriptive statistics

Table 1 presents the overall characteristics of the 97,481 respondents sampled from 1999 to 2014. Over the period, the crude overall prevalence of self-reported physical-diagnosed diabetes increased substantially in both genders (see Additional file 1, Additional file 2 and Additional file 3).

**Table 1:** Basic overall characteristics of all respondents between 1999 and 2014 (*N* = 97,481)

	Female		Male	
	*N*	(Column %)	*N*	(Column %)
*Age*				
45–49	10,649	(21.5%)	9939	(20.7%)
50–54	9733	(19.7%)	9828	(20.5%)
55–59	6778	(13.7%)	7055	(14.7%)
60–64	5774	(11.7%)	6017	(12.5%)
65 or above	16,567	(33.5%)	15,141	(31.6%)
*Marital status*				
Married	33,971	(68.6%)	41,132	(85.7%)
Non-married	15,513	(31.3%)	6833	(14.2%)
Missing	17	(0.0%)	15	(0.0%)
*Household size*				
1	4287	(8.7%)	3865	(8.1%)
2	11,721	(23.7%)	9731	(20.3%)
3	12,467	(25.2%)	12,165	(25.4%)
4	12,234	(24.7%)	13,640	(28.4%)
5 or above	8792	(17.8%)	8579	(17.9%)
*Education*				
Below primary level	10,664	(21.5%)	4166	(8.7%)
Primary level	17,263	(34.9%)	16,069	(33.5%)
Secondary level	18,298	(37.0%)	22,257	(46.4%)
Tertiary level	3257	(6.6%)	5471	(11.4%)
Missing	19	(0.0%)	17	(0.0%)
*Household income (HKD)*				
$9999 or less	13,474	(27.2%)	12,278	(25.6%)
$10,000–24,999	17,894	(36.1%)	18,113	(37.8%)
$25,000–49,999	12,550	(25.4%)	12,067	(25.2%)
$50,000 or above	4747	(9.6%)	4763	(9.9%)
Missing	836	(1.7%)	759	(1.6%)
*Diabetes*				
No	45,200	(91.3%)	44,025	(91.8%)
Yes	4301	(8.7%)	3955	(8.2%)
*Survey year*				
1999	5251	(10.6%)	5294	(11.0%)
2001	5603	(11.3%)	5625	(11.7%)
2002	5355	(10.8%)	5317	(11.1%)
2005	5994	(12.1%)	6031	(12.6%)
2008	6332	(12.8%)	6319	(13.2%)
2009	6667	(13.5%)	6336	(13.2%)
2011	7022	(14.2%)	6435	(13.4%)
2014	7277	(14.7%)	6623	(13.8%)

### Age-standardized prevalence of diabetes

**Fig. 1:**
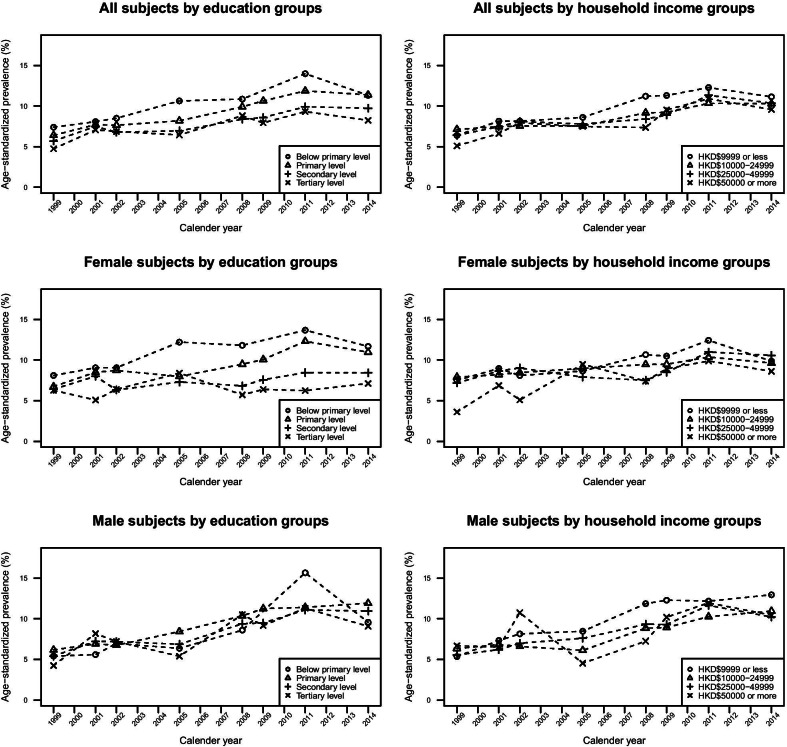
Trends of overall and gender-specific prevalence of diabetes by education and household income groups

The total and gender-specific age-standardized prevalence of self-reported diabetes across education and household income groups is presented in Fig. 1. The prevalence of diabetes across education and household income groups was similar in earlier years of our study. Nonetheless, the extent of increase in diabetes prevalence differed by education groups and genders. In female, a more apparent rise in diabetes was found among the less educated women, whereas the steep increase among men was non-differential across education levels. In general, the increase in prevalence of diabetes did not deviate much across household income levels in both genders.

### Relative and absolute educational inequalities in diabetes

**Fig. 2:**
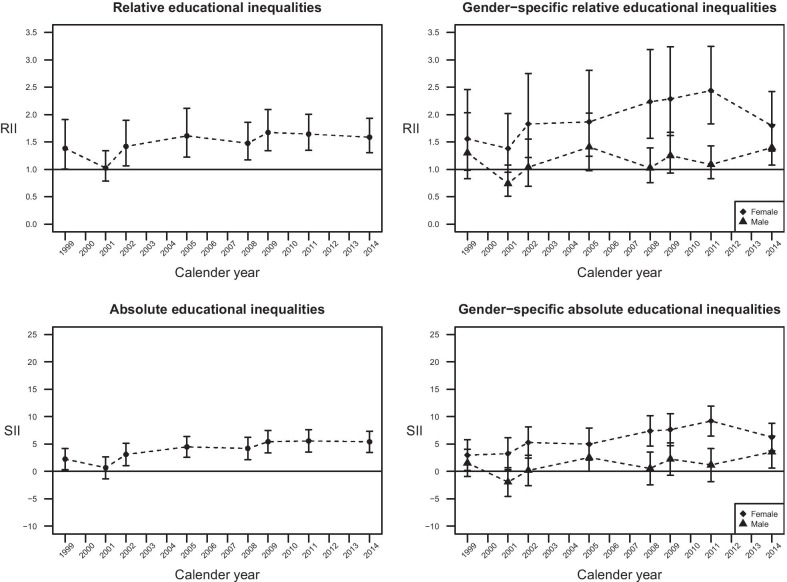
Trends of overall and gender-specific relative and absolute educational inequalities in diabetes

Figure 2 presents the overall and gender-specific relative and absolute educational inequalities in diabetes across survey years. While increasing RIIs and SIIs were observed in the total sample, the widening inequality in diabetes was apparent only in female but not in male. In female, RIIs and SIIs rose from 1.56 (95% CI = 0.99–2.46) to 1.80 (95% CI = 1.33–2.42) and from 2.99% (95% CI = 0.15–5.83%) to 6.26% (95% CI = 3.73–8.79%), respectively, between 1999 and 2014, with the peak in 2011 (RII = 2.44; 95% CI = 1.83–3.24, SII = 9.21%; 95% CI = 6.47–11.96%). The annual changes in RII and SII in female were estimated to be 1.04 (95% CI = 1.02–1.07) and 0.36% (95% CI = 0.16–0.56%), respectively. However, no significant relative or absolute inequality across years was found in male (annual RII change = 1.01; 95% CI = 0.99–1.04, annual SII change = 0.17; 95% CI =  − 0.03 to 0.37), except in 2005 and 2014. Further details on the RII and SII measures across survey years are available (see Additional file 4).

### Educational inequalities after adjusting for household income

Educational inequalities after further adjustment for household income levels across survey years are also presented (see Additional file 5). The extent of educational inequality was not significantly attenuated by household income level, and the overall patterns were consistent with the results shown in Fig. 2.

## Discussion

### Summary of findings

A surge in age-standardized prevalence of diabetes was observed in both genders, with a greater leap among men, between 1999 and 2014. Also, we found significant gender-specific differences in these secular trends across educational levels. Despite a potential reduction in inequality after 2011, the educational gradient in diabetes increased apparently over time among women; i.e., the less educated the women, the greater the secular increase in diabetes prevalence was. In particular, the discrepancies across education levels among women increased at an annual rate of 4% relatively and 0.36% absolutely over the study period. In addition, the observed educational inequality was independent of the respondents’ household income levels. Nonetheless, no evident educational patterning and apparent trend were observed among men.

### Greater surge in diabetes among men relative to women

Over the recent years, male gender has been associated with diabetes, especially in developed world regions [27–29]. Hong Kong is no exception to such a pattern. Local research reported higher incidence and greater increase in prevalence of diabetes among men over the last decade [30], which echoed our finding on the gender-specific surge in diagnosed diabetes. The stronger prevalence in male was found to be associated with the greater amount of visceral fat among men relative to women [31]. Nonetheless, our findings further attributed the gender-specific surge in diabetes to the differential impacts of education on diabetes between genders. The lower surge among women in Hong Kong was due to the less apparent increase among more educated women relative to their less educated counterparts, given that the steep rise among men was non-differential across education levels.

### Gender difference in educational inequality in diabetes

Consistent with our findings, a more pronounced educational inequality in diabetes has been commonly observed among women [8, 14, 17, 32, 33]. The gender-specific impacts of education on diabetes may operate through obesity, since the educational gradient in obesity among women was found stronger when compared to men [34, 35]. In general, highly educated women adopt stricter behavioral norms for slimness than men and less educated female counterparts [36]. The impact of obesity among women may thus lead to differential incidence rates across education levels, given that a recent systematic review concluded a stronger educational inequality in incident diabetes among women relative to men [9]. Furthermore, the gender-specific trends could have plausibly been driven by the differential responsiveness to these health information between genders [37] and coping capability across education levels [38]. In terms of responsiveness, being female and more educated tends to seek health information more frequently through a range of communication channels [37, 39]. While highly educated women are expected to benefit the most from the increasingly overwhelming health information, their less educated counterparts tend to experience negative emotions due to maladaptive coping to similar health information and thus have a reduced behavioral intention to cardio-protective behaviors [38, 40]. Therefore, along with the progressively abundant health information, the increase in diabetes prevalence among women was the greatest in the least educated but the least in the most educated, whereas that among men was similar across education levels as men are generally less responsive to the health information [37]. Despite the apparent widening trend between 1999 and 2011 among women, we observed a potential narrowing educational inequality in diabetes between 2011 and 2014. While it is possibly a chance finding, a downward trend over similar period has also been reported by a recent study in South Korea [20]. Continuous monitoring and investigations are thus warranted to verify the potential recent decline in educational inequality in diabetes in Hong Kong and other Asian economies.

It is also worth noting that our study found that the observed trends of educational inequality could not be explained by household income level. While household income may not be the best indicator to reflect an individual’s poverty status and capture its link to health [41, 42], the negligible attenuation of educational inequality by household income level suggests that cost-related mechanisms are insufficient to explain the widening educational inequality among women. Rather, pathways through health literacy and lifestyle appear to be important drivers of the educational difference in diabetes among women.

### Public health implications

The rising diabetes burden among men and less educated women would bring enormous challenges on their self-management and also on the healthcare system in Hong Kong. Policy makers should consider the gender differences in designing future prevention and intervention strategies. While a universal approach should be adopted for men to control the overall surge, extra effort may be given to less educated women to reduce the extent of educational inequality in diabetes. With less knowledge and lower coping capability, the less educated women could hardly take advantage of the increasingly effective but complicated diabetes management [43]. Also, previous research suggested a gender difference in self-management that women with diabetes tend to be less physically active, have less frequent self-monitoring of blood glucose, and experience greater socioeconomic barriers and psychological distress when compared to men [44]. With the surge in diabetes but relatively poor self-management, the less educated women may become the most vulnerable group to diabetes-related complications in the coming decades. Moreover, in light of their greater psychological needs, screening programs for subclinical psychiatric disorders may be warranted for less educated diabetic women, and so do the associated mental health services.

### Strengths and limitations

The present study is the first to examine gender-specific trends of educational inequality in diabetes in a series of representative samples of Hong Kong Chinese adults, which echoed previous studies in developed world regions and enriched the limited research in Asia. However, there are several caveats in our study. Firstly, we relied on self-reported data of physician-diagnosed diabetes since clinical measurements of plasma glucose level were not available. Thus, the increase in self-reported physician-diagnosed diabetes over time may be affected by increased reporting due to overall improvement in health literacy [45]. Also, as underreporting of diseases may be more common among the disadvantaged group [46], the actual educational inequality may be even wider in reality. Nonetheless, our main finding of a widening educational inequality in diabetes among women is more likely a result of increased incidence rather than of reporting issues, given that a stronger social gradient in incidence of diabetes is commonly observed among women relative to men in developed settings [8, 32]. Moreover, previous research confirmed reasonable sensitivity and specificity of self-reported physician-diagnosed diabetes obtained via health interview surveys to assess social inequalities in prevalence of diabetes [47]. Secondly, although educational attainment and household income are generally deemed as the conventional socioeconomic indicators, they may not fully capture the health impact of other dimensions of socioeconomic disadvantage such as occupation [48, 49], housing [50] and material and social deprivation [41, 42]. The potential underreporting of income level in surveys may also introduce bias to the nonsignificant association of household income with diabetes prevalence observed in this study. Nonetheless, a recent systematic review on socioeconomic status and diabetes among ethnic Chinese supported that low educational attainment is the most consistent socioeconomic indicator of an increased diabetes prevalence when compared to income and occupation [51]. Thirdly, due to data availability, potential confounders including medical history and genetic differences were not adjusted. It is also worth noting that nutritional outcomes such as obesity, which are also socially patterned, should not be adjusted for the purpose of confounding control. The adjustments for them will attenuate the total effect of education and thus is tangential to our main objective to assess the disparities in diabetes across education levels. Lastly, RIIs and SIIs should be compared with cautions. As age standardization was only applied to SIIs but not to RIIs, directions of estimates of the two complementary measures in a given year may be different if the association is close to null; however, the two estimates in the same year would be in the same direction whenever a statistically significant educational inequality was observed.

## Conclusion

Over the recent two decades, the surge in diabetes was greater among men; nonetheless, the discrepancy in prevalence widened apparently across educational levels among women. Tremendous treatment needs among men and, to an even greater extent, among the less educated women are expected in the near future. To tackle the educational inequality in diabetes, policy makers should go beyond endowment and consider the gender perspective as well as its interaction with social disadvantages when designing prevention and intervention strategies.

## Supplementary Information

**Additional file 1.** Basic characteristics of all respondents (N = 97,481). Over descriptive statistics of respondents stratified by 8 survey years.

**Additional file 2.** Basic characteristics of female respondents (N = 49,501). Descriptive statistics of female respondents stratified by 8 survey years.

**Additional file 3.** Basic characteristics of male respondents (N=47,980). Descriptive statistics of male respondents stratified by 8 survey years.

**Additional file 4.** Relative and absolute educational inequalities in diabetes across years. RII and SII measures across 8 survey years.

**Additional file 5.** Relative and absolute educational inequalities in diabetes across years after further adjustments for household income. RII and SII measures across 8 survey years after adjustment for household income level.

## Data Availability

The data that support the findings of this study are available from C&SD of the Hong Kong Government, but restrictions apply to the availability of these data, which were used under license for the current study, and so are not publicly available. Data are, however, available from the authors upon reasonable request and with permission of C&SD of the Hong Kong Government.

## Abbreviations

C&SD: Census and Statistics Department
CI: Confidence interval
HKD: Hong Kong Dollar
OR: Odds ratio
RII: Relative Index of Inequality
SII: Slope Index of Inequality
THS: Thematic Household Survey
USD: US Dollar
