# Supplementary material for: Gender-specific trends of educational inequality in diagnosed diabetes from 1999 to 2014 in Hong Kong: a serial cross-sectional study of 97,481 community-dwelling Chinese adults
Source: Popul Health Metr. 2021 Oct 10;19:37. doi: 10.1186/s12963-021-00268-x (PMC8504033; doi:10.1186/s12963-021-00268-x)
Supplement: Supplementary file 1 — Additional file 1. Basic characteristics of all respondents (N = 97,481). Over descriptive statistics of respondents stratified by 8 survey years. [file 12963_2021_268_MOESM1_ESM.docx]

| **Additional file 1. Basic characteristics of all respondents (N=97,481)** | | | | | | | | | | | | | | | | | | | | | | | | | | | | |
| --- | --- | --- | --- | --- | --- | --- | --- | --- | --- | --- | --- | --- | --- | --- | --- | --- | --- | --- | --- | --- | --- | --- | --- | --- | --- | --- | --- | --- |
|  |  |  | 1999 | |  | 2001 | |  | 2002 | |  | 2005 | |  | 2008 | |  | 2009 | |  | 2011 | |  | 2014 | |  | Total | |
|  |  |  | N | (Column %) |  | N | (Column %) |  | N | (Column %) |  | N | (Column %) |  | N | (Column %) |  | N | (Column %) |  | N | (Column %) |  | N | (Column %) |  | N | (Column %) |
| **All** | |  | 10,545 |  |  | 11,228 |  |  | 10,672 |  |  | 12,025 |  |  | 12,651 |  |  | 13,003 |  |  | 13,457 |  |  | 13,900 |  |  | 97,481 |  |
|  | *Age* | |  |  |  |  |  |  |  |  |  |  |  |  |  |  |  |  |  |  |  |  |  |  |  |  |  |  |
|  |  | 45-49 | 2,441 | (23.1%) |  | 2,571 | (22.9%) |  | 2,443 | (22.9%) |  | 2,842 | (23.6%) |  | 2,730 | (21.6%) |  | 2,670 | (20.5%) |  | 2,617 | (19.4%) |  | 2,274 | (16.4%) |  | 20,588 | (21.1%) |
|  |  | 50-54 | 1,979 | (18.8%) |  | 2,285 | (20.4%) |  | 2,063 | (19.3%) |  | 2,504 | (20.8%) |  | 2,595 | (20.5%) |  | 2,693 | (20.7%) |  | 2,700 | (20.1%) |  | 2,742 | (19.7%) |  | 19,561 | (20.1%) |
|  |  | 55-59 | 1,155 | (11.0%) |  | 1,252 | (11.2%) |  | 1,296 | (12.1%) |  | 1,749 | (14.5%) |  | 1,899 | (15.0%) |  | 1,938 | (14.9%) |  | 2,176 | (16.2%) |  | 2,368 | (17.0%) |  | 13,833 | (14.2%) |
|  |  | 60-64 | 1,359 | (12.9%) |  | 1,241 | (11.1%) |  | 1,167 | (10.9%) |  | 1,190 | (9.9%) |  | 1,385 | (10.9%) |  | 1,685 | (13.0%) |  | 1,760 | (13.1%) |  | 2,004 | (14.4%) |  | 11,791 | (12.1%) |
|  |  | 65 or above | 3,611 | (34.2%) |  | 3,879 | (34.5%) |  | 3,703 | (34.7%) |  | 3,740 | (31.1%) |  | 4,042 | (32.0%) |  | 4,017 | (30.9%) |  | 4,204 | (31.2%) |  | 4,512 | (32.5%) |  | 31,708 | (32.5%) |
|  | *Gender* | |  |  |  |  |  |  |  |  |  |  |  |  |  |  |  |  |  |  |  |  |  |  |  |  |  |  |
|  |  | Female | 5,251 | (49.8%) |  | 5,603 | (49.9%) |  | 5,355 | (50.2%) |  | 5,994 | (49.8%) |  | 6,332 | (50.1%) |  | 6,667 | (51.3%) |  | 7,022 | (52.2%) |  | 7,277 | (52.4%) |  | 49,501 | (50.8%) |
|  |  | Male | 5,294 | (50.2%) |  | 5,625 | (50.1%) |  | 5,317 | (49.8%) |  | 6,031 | (50.2%) |  | 6,319 | (49.9%) |  | 6,336 | (48.7%) |  | 6,435 | (47.8%) |  | 6,623 | (47.6%) |  | 47,980 | (49.2%) |
|  | *Marital status* | |  |  |  |  |  |  |  |  |  |  |  |  |  |  |  |  |  |  |  |  |  |  |  |  |  |  |
|  |  | Married | 8,237 | (78.1%) |  | 9,239 | (82.3%) |  | 8,233 | (77.1%) |  | 9,459 | (78.7%) |  | 9,613 | (76.0%) |  | 9,881 | (76.0%) |  | 10,067 | (74.8%) |  | 10,374 | (74.6%) |  | 75,103 | (77.0%) |
|  |  | Non-married | 2,283 | (21.7%) |  | 1,989 | (17.7%) |  | 2,432 | (22.8%) |  | 2,566 | (21.3%) |  | 3,038 | (24.0%) |  | 3,122 | (24.0%) |  | 3,390 | (25.2%) |  | 3,526 | (25.4%) |  | 22,346 | (22.9%) |
|  |  | Missing | 25 | (0.2%) |  | 0 | (0.0%) |  | 7 | (0.1%) |  | 0 | (0.0%) |  | 0 | (0.0%) |  | 0 | (0.0%) |  | 0 | (0.0%) |  | 0 | (0.0%) |  | 32 | (0.0%) |
|  | *Household size* | |  |  |  |  |  |  |  |  |  |  |  |  |  |  |  |  |  |  |  |  |  |  |  |  |  |  |
|  |  | 1 | 774 | (7.3%) |  | 655 | (5.8%) |  | 1,069 | (10.0%) |  | 897 | (7.5%) |  | 1,110 | (8.8%) |  | 1,177 | (9.1%) |  | 1,233 | (9.2%) |  | 1,237 | (8.9%) |  | 8,152 | (8.4%) |
|  |  | 2 | 1,890 | (17.9%) |  | 1,941 | (17.3%) |  | 2,411 | (22.6%) |  | 2,587 | (21.5%) |  | 2,863 | (22.6%) |  | 3,123 | (24.0%) |  | 3,125 | (23.2%) |  | 3,512 | (25.3%) |  | 21,452 | (22.0%) |
|  |  | 3 | 2,166 | (20.5%) |  | 2,498 | (22.2%) |  | 2,572 | (24.1%) |  | 3,191 | (26.5%) |  | 3,338 | (26.4%) |  | 3,474 | (26.7%) |  | 3,643 | (27.1%) |  | 3,750 | (27.0%) |  | 24,632 | (25.3%) |
|  |  | 4 | 2,871 | (27.2%) |  | 3,413 | (30.4%) |  | 2,640 | (24.7%) |  | 3,229 | (26.9%) |  | 3,350 | (26.5%) |  | 3,354 | (25.8%) |  | 3,516 | (26.1%) |  | 3,501 | (25.2%) |  | 25,874 | (26.5%) |
|  |  | 5 or above | 2,844 | (27.0%) |  | 2,721 | (24.2%) |  | 1,980 | (18.6%) |  | 2,121 | (17.6%) |  | 1,990 | (15.7%) |  | 1,875 | (14.4%) |  | 1,940 | (14.4%) |  | 1,900 | (13.7%) |  | 17,371 | (17.8%) |
|  | *Education* | |  |  |  |  |  |  |  |  |  |  |  |  |  |  |  |  |  |  |  |  |  |  |  |  |  |  |
|  |  | Below primary level | 2,486 | (23.6%) |  | 2,226 | (19.8%) |  | 2,341 | (21.9%) |  | 1,895 | (15.8%) |  | 1,634 | (12.9%) |  | 1,435 | (11.0%) |  | 1,448 | (10.8%) |  | 1,365 | (9.8%) |  | 14,830 | (15.2%) |
|  |  | Primary level | 3,951 | (37.5%) |  | 4,386 | (39.1%) |  | 3,872 | (36.3%) |  | 4,066 | (33.8%) |  | 4,403 | (34.8%) |  | 4,324 | (33.3%) |  | 4,132 | (30.7%) |  | 4,198 | (30.2%) |  | 33,332 | (34.2%) |
|  |  | Secondary level | 3,325 | (31.5%) |  | 3,805 | (33.9%) |  | 3,629 | (34.0%) |  | 5,021 | (41.8%) |  | 5,537 | (43.8%) |  | 6,048 | (46.5%) |  | 6,454 | (48.0%) |  | 6,736 | (48.5%) |  | 40,555 | (41.6%) |
|  |  | Tertiary level | 760 | (7.2%) |  | 811 | (7.2%) |  | 817 | (7.7%) |  | 1,043 | (8.7%) |  | 1,077 | (8.5%) |  | 1,196 | (9.2%) |  | 1,423 | (10.6%) |  | 1,601 | (11.5%) |  | 8,728 | (9.0%) |
|  |  | Missing | 23 | (0.2%) |  | 0 | (0.0%) |  | 13 | (0.1%) |  | 0 | (0.0%) |  | 0 | (0.0%) |  | 0 | (0.0%) |  | 0 | (0.0%) |  | 0 | (0.0%) |  | 36 | (0.0%) |
|  | *Household income (HKD)* | |  |  |  |  |  |  |  |  |  |  |  |  |  |  |  |  |  |  |  |  |  |  |  |  |  |  |
|  |  | $9999 or less | 2,906 | (27.6%) |  | 3,124 | (27.8%) |  | 3,414 | (32.0%) |  | 2,927 | (24.3%) |  | 3,572 | (28.2%) |  | 3,837 | (29.5%) |  | 3,182 | (23.6%) |  | 2,790 | (20.1%) |  | 25,752 | (26.4%) |
|  |  | $10000-24999 | 3,837 | (36.4%) |  | 4,140 | (36.9%) |  | 3,838 | (36.0%) |  | 4,914 | (40.9%) |  | 4,828 | (38.2%) |  | 5,018 | (38.6%) |  | 4,806 | (35.7%) |  | 4,626 | (33.3%) |  | 36,007 | (36.9%) |
|  |  | $25000-49999 | 2,325 | (22.0%) |  | 2,829 | (25.2%) |  | 2,034 | (19.1%) |  | 3,054 | (25.4%) |  | 3,003 | (23.7%) |  | 2,928 | (22.5%) |  | 3,990 | (29.6%) |  | 4,454 | (32.0%) |  | 24,617 | (25.3%) |
|  |  | $50000 or above | 902 | (8.6%) |  | 1,135 | (10.1%) |  | 665 | (6.2%) |  | 831 | (6.9%) |  | 1,248 | (9.9%) |  | 1,220 | (9.4%) |  | 1,479 | (11.0%) |  | 2,030 | (14.6%) |  | 9,510 | (9.8%) |
|  |  | Missing | 575 | (5.5%) |  | 0 | (0.0%) |  | 721 | (6.8%) |  | 299 | (2.5%) |  | 0 | (0.0%) |  | 0 | (0.0%) |  | 0 | (0.0%) |  | 0 | (0.0%) |  | 1,595 | (1.6%) |
|  | *Diabetes* | |  |  |  |  |  |  |  |  |  |  |  |  |  |  |  |  |  |  |  |  |  |  |  |  |  |  |
|  |  | No | 9,897 | (93.9%) |  | 10,433 | (92.9%) |  | 9,909 | (92.9%) |  | 11,158 | (92.8%) |  | 11,522 | (91.1%) |  | 11,794 | (90.7%) |  | 12,033 | (89.4%) |  | 12,479 | (89.8%) |  | 89,225 | (91.5%) |
|  |  | Yes | 648 | (6.1%) |  | 795 | (7.1%) |  | 763 | (7.1%) |  | 867 | (7.2%) |  | 1,129 | (8.9%) |  | 1,209 | (9.3%) |  | 1,424 | (10.6%) |  | 1,421 | (10.2%) |  | 8,256 | (8.5%) |
