# Supplementary material for: Gender-specific trends of educational inequality in diagnosed diabetes from 1999 to 2014 in Hong Kong: a serial cross-sectional study of 97,481 community-dwelling Chinese adults
Source: Popul Health Metr. 2021 Oct 10;19:37. doi: 10.1186/s12963-021-00268-x (PMC8504033; doi:10.1186/s12963-021-00268-x)
Supplement: Supplementary file 2 — Additional file 2. Basic characteristics of female respondents (N = 49,501). Descriptive statistics of female respondents stratified by 8 survey years. [file 12963_2021_268_MOESM2_ESM.docx]

| **Additional file 2. Basic characteristics of female respondents (N=49,501)** | | | | | | | | | | | | | | | | | | | | | | | | | | | | |
| --- | --- | --- | --- | --- | --- | --- | --- | --- | --- | --- | --- | --- | --- | --- | --- | --- | --- | --- | --- | --- | --- | --- | --- | --- | --- | --- | --- | --- |
|  |  |  | 1999 | |  | 2001 | |  | 2002 | |  | 2005 | |  | 2008 | |  | 2009 | |  | 2011 | |  | 2014 | |  | Total | |
|  |  |  | N | (Column %) |  | N | (Column %) |  | N | (Column %) |  | N | (Column %) |  | N | (Column %) |  | N | (Column %) |  | N | (Column %) |  | N | (Column %) |  | N | (Column %) |
| **Female** | | | 5,251 |  |  | 5,603 |  |  | 5,355 |  |  | 5,994 |  |  | 6,332 |  |  | 6,667 |  |  | 7,022 |  |  | 7,277 |  |  | 49,501 |  |
|  | *Age* | |  |  |  |  |  |  |  |  |  |  |  |  |  |  |  |  |  |  |  |  |  |  |  |  |  |  |
|  |  | 45-49 | 1,208 | (23.0%) |  | 1,295 | (23.1%) |  | 1,242 | (23.2%) |  | 1,423 | (23.7%) |  | 1,360 | (21.5%) |  | 1,434 | (21.5%) |  | 1,455 | (20.7%) |  | 1,232 | (16.9%) |  | 10,649 | (21.5%) |
|  |  | 50-54 | 953 | (18.1%) |  | 1,096 | (19.6%) |  | 991 | (18.5%) |  | 1,276 | (21.3%) |  | 1,297 | (20.5%) |  | 1,326 | (19.9%) |  | 1,364 | (19.4%) |  | 1,430 | (19.7%) |  | 9,733 | (19.7%) |
|  |  | 55-59 | 503 | (9.6%) |  | 578 | (10.3%) |  | 640 | (12.0%) |  | 830 | (13.8%) |  | 929 | (14.7%) |  | 983 | (14.7%) |  | 1,073 | (15.3%) |  | 1,242 | (17.1%) |  | 6,778 | (13.7%) |
|  |  | 60-64 | 639 | (12.2%) |  | 594 | (10.6%) |  | 529 | (9.9%) |  | 562 | (9.4%) |  | 666 | (10.5%) |  | 846 | (12.7%) |  | 907 | (12.9%) |  | 1,031 | (14.2%) |  | 5,774 | (11.7%) |
|  |  | 65 or above | 1,948 | (37.1%) |  | 2,040 | (36.4%) |  | 1,953 | (36.5%) |  | 1,903 | (31.7%) |  | 2,080 | (32.8%) |  | 2,078 | (31.2%) |  | 2,223 | (31.7%) |  | 2,342 | (32.2%) |  | 16,567 | (33.5%) |
|  | *Marital status* | |  |  |  |  |  |  |  |  |  |  |  |  |  |  |  |  |  |  |  |  |  |  |  |  |  |  |
|  |  | Married | 3,616 | (68.9%) |  | 4,222 | (75.4%) |  | 3,655 | (68.3%) |  | 4,274 | (71.3%) |  | 4,308 | (68.0%) |  | 4,473 | (67.1%) |  | 4,617 | (65.8%) |  | 4,806 | (66.0%) |  | 33,971 | (68.6%) |
|  |  | Non-married | 1,624 | (30.9%) |  | 1,381 | (24.6%) |  | 1,694 | (31.6%) |  | 1,720 | (28.7%) |  | 2,024 | (32.0%) |  | 2,194 | (32.9%) |  | 2,405 | (34.2%) |  | 2,471 | (34.0%) |  | 15,513 | (31.3%) |
|  |  | Missing | 11 | (0.2%) |  | 0 | (0.0%) |  | 6 | (0.1%) |  | 0 | (0.0%) |  | 0 | (0.0%) |  | 0 | (0.0%) |  | 0 | (0.0%) |  | 0 | (0.0%) |  | 17 | (0.0%) |
|  | *Household size* | |  |  |  |  |  |  |  |  |  |  |  |  |  |  |  |  |  |  |  |  |  |  |  |  |  |  |
|  |  | 1 | 374 | (7.1%) |  | 324 | (5.8%) |  | 541 | (10.1%) |  | 448 | (7.5%) |  | 539 | (8.5%) |  | 643 | (9.6%) |  | 716 | (10.2%) |  | 702 | (9.6%) |  | 4,287 | (8.7%) |
|  |  | 2 | 1,024 | (19.5%) |  | 1,049 | (18.7%) |  | 1,282 | (23.9%) |  | 1,394 | (23.3%) |  | 1,562 | (24.7%) |  | 1,720 | (25.8%) |  | 1,735 | (24.7%) |  | 1,955 | (26.9%) |  | 11,721 | (23.7%) |
|  |  | 3 | 1,071 | (20.4%) |  | 1,288 | (23.0%) |  | 1,317 | (24.6%) |  | 1,582 | (26.4%) |  | 1,666 | (26.3%) |  | 1,775 | (26.6%) |  | 1,865 | (26.6%) |  | 1,903 | (26.2%) |  | 12,467 | (25.2%) |
|  |  | 4 | 1,338 | (25.5%) |  | 1,572 | (28.1%) |  | 1,226 | (22.9%) |  | 1,512 | (25.2%) |  | 1,574 | (24.9%) |  | 1,577 | (23.7%) |  | 1,697 | (24.2%) |  | 1,738 | (23.9%) |  | 12,234 | (24.7%) |
|  |  | 5 or above | 1,444 | (27.5%) |  | 1,370 | (24.5%) |  | 989 | (18.5%) |  | 1,058 | (17.7%) |  | 991 | (15.7%) |  | 952 | (14.3%) |  | 1,009 | (14.4%) |  | 979 | (13.5%) |  | 8,792 | (17.8%) |
|  | *Education* | |  |  |  |  |  |  |  |  |  |  |  |  |  |  |  |  |  |  |  |  |  |  |  |  |  |  |
|  |  | Below primary level | 1,792 | (34.1%) |  | 1,609 | (28.7%) |  | 1,633 | (30.5%) |  | 1,319 | (22.0%) |  | 1,172 | (18.5%) |  | 1,050 | (15.7%) |  | 1,066 | (15.2%) |  | 1,023 | (14.1%) |  | 10,664 | (21.5%) |
|  |  | Primary level | 1,881 | (35.8%) |  | 2,168 | (38.7%) |  | 1,917 | (35.8%) |  | 2,078 | (34.7%) |  | 2,309 | (36.5%) |  | 2,331 | (35.0%) |  | 2,268 | (32.3%) |  | 2,311 | (31.8%) |  | 17,263 | (34.9%) |
|  |  | Secondary level | 1,315 | (25.0%) |  | 1,534 | (27.4%) |  | 1,522 | (28.4%) |  | 2,257 | (37.7%) |  | 2,454 | (38.8%) |  | 2,812 | (42.2%) |  | 3,129 | (44.6%) |  | 3,275 | (45.0%) |  | 18,298 | (37.0%) |
|  |  | Tertiary level | 252 | (4.8%) |  | 292 | (5.2%) |  | 275 | (5.1%) |  | 340 | (5.7%) |  | 397 | (6.3%) |  | 474 | (7.1%) |  | 559 | (8.0%) |  | 668 | (9.2%) |  | 3,257 | (6.6%) |
|  |  | Missing | 11 | (0.2%) |  | 0 | (0.0%) |  | 8 | (0.1%) |  | 0 | (0.0%) |  | 0 | (0.0%) |  | 0 | (0.0%) |  | 0 | (0.0%) |  | 0 | (0.0%) |  | 19 | (0.0%) |
|  | *Household income (HKD)* | |  |  |  |  |  |  |  |  |  |  |  |  |  |  |  |  |  |  |  |  |  |  |  |  |  |  |
|  |  | $9999 or less | 1,451 | (27.6%) |  | 1,584 | (28.3%) |  | 1,718 | (32.1%) |  | 1,505 | (25.1%) |  | 1,844 | (29.1%) |  | 2,041 | (30.6%) |  | 1,772 | (25.2%) |  | 1,559 | (21.4%) |  | 13,474 | (27.2%) |
|  |  | $10000-24999 | 1,849 | (35.2%) |  | 1,995 | (35.6%) |  | 1,919 | (35.8%) |  | 2,394 | (39.9%) |  | 2,366 | (37.4%) |  | 2,520 | (37.8%) |  | 2,458 | (35.0%) |  | 2,393 | (32.9%) |  | 17,894 | (36.1%) |
|  |  | $25000-49999 | 1,181 | (22.5%) |  | 1,447 | (25.8%) |  | 1,033 | (19.3%) |  | 1,532 | (25.6%) |  | 1,508 | (23.8%) |  | 1,495 | (22.4%) |  | 2,052 | (29.2%) |  | 2,302 | (31.6%) |  | 12,550 | (25.4%) |
|  |  | $50000 or above | 458 | (8.7%) |  | 577 | (10.3%) |  | 318 | (5.9%) |  | 406 | (6.8%) |  | 614 | (9.7%) |  | 611 | (9.2%) |  | 740 | (10.5%) |  | 1,023 | (14.1%) |  | 4,747 | (9.6%) |
|  |  | Missing | 312 | (5.9%) |  | 0 | (0.0%) |  | 367 | (6.9%) |  | 157 | (2.6%) |  | 0 | (0.0%) |  | 0 | (0.0%) |  | 0 | (0.0%) |  | 0 | (0.0%) |  | 836 | (1.7%) |
|  | *Diabetes* | |  |  |  |  |  |  |  |  |  |  |  |  |  |  |  |  |  |  |  |  |  |  |  |  |  |  |
|  |  | No | 4,882 | (93.0%) |  | 5,163 | (92.1%) |  | 4,935 | (92.2%) |  | 5,530 | (92.3%) |  | 5,775 | (91.2%) |  | 6,062 | (90.9%) |  | 6,275 | (89.4%) |  | 6,578 | (90.4%) |  | 45,200 | (91.3%) |
|  |  | Yes | 369 | (7.0%) |  | 440 | (7.9%) |  | 420 | (7.8%) |  | 464 | (7.7%) |  | 557 | (8.8%) |  | 605 | (9.1%) |  | 747 | (10.6%)  ) |  | 699 | (9.6%) |  | 4,301 | (8.7%) |
