# Supplementary material for: Gender-specific trends of educational inequality in diagnosed diabetes from 1999 to 2014 in Hong Kong: a serial cross-sectional study of 97,481 community-dwelling Chinese adults
Source: Popul Health Metr. 2021 Oct 10;19:37. doi: 10.1186/s12963-021-00268-x (PMC8504033; doi:10.1186/s12963-021-00268-x)
Supplement: Supplementary file 3 — Additional file 3. Basic characteristics of male respondents (N=47,980). Descriptive statistics of male respondents stratified by 8 survey years. [file 12963_2021_268_MOESM3_ESM.docx]

| **Additional file 3.** **Basic characteristics of male respondents (N=47,980)** | | | | | | | | | | | | | | | | | | | | | | | | | | | | |
| --- | --- | --- | --- | --- | --- | --- | --- | --- | --- | --- | --- | --- | --- | --- | --- | --- | --- | --- | --- | --- | --- | --- | --- | --- | --- | --- | --- | --- |
|  |  |  | 1999 | |  | 2001 | |  | 2002 | |  | 2005 | |  | 2008 | |  | 2009 | |  | 2011 | |  | 2014 | |  | Total | |
|  |  |  | N | (Column %) |  | N | (Column %) |  | N | (Column %) |  | N | (Column %) |  | N | (Column %) |  | N | (Column %) |  | N | (Column %) |  | N | (Column %) |  | N | (Column %) |
| **Male** | | | 5,294 |  |  | 5,625 |  |  | 5,317 |  |  | 6,031 |  |  | 6,319 |  |  | 6,336 |  |  | 6,435 |  |  | 6,623 |  |  | 47,980 |  |
|  | *Age* | |  |  |  |  |  |  |  |  |  |  |  |  |  |  |  |  |  |  |  |  |  |  |  |  |  |  |
|  |  | 45-49 | 1,233 | (23.3%) |  | 1,276 | (22.7%) |  | 1,201 | (22.6%) |  | 1,419 | (23.5%) |  | 1,370 | (21.7%) |  | 1,236 | (19.5%) |  | 1,162 | (18.1%) |  | 1,042 | (15.7%) |  | 9,939 | (20.7%) |
|  |  | 50-54 | 1,026 | (19.4%) |  | 1,189 | (21.1%) |  | 1,072 | (20.2%) |  | 1,228 | (20.4%) |  | 1,298 | (20.5%) |  | 1,367 | (21.6%) |  | 1,336 | (20.8%) |  | 1,312 | (19.8%) |  | 9,828 | (20.5%) |
|  |  | 55-59 | 652 | (12.3%) |  | 674 | (12.0%) |  | 656 | (12.3%) |  | 919 | (15.2%) |  | 970 | (15.4%) |  | 955 | (15.1%) |  | 1,103 | (17.1%) |  | 1,126 | (17.0%) |  | 7,055 | (14.7%) |
|  |  | 60-64 | 720 | (13.6%) |  | 647 | (11.5%) |  | 638 | (12.0%) |  | 628 | (10.4%) |  | 719 | (11.4%) |  | 839 | (13.2%) |  | 853 | (13.3%) |  | 973 | (14.7%) |  | 6,017 | (12.5%) |
|  |  | 65 or above | 1,663 | (31.4%) |  | 1,839 | (32.7%) |  | 1,750 | (32.9%) |  | 1,837 | (30.5%) |  | 1,962 | (31.0%) |  | 1,939 | (30.6%) |  | 1,981 | (30.8%) |  | 2,170 | (32.8%) |  | 15,141 | (31.6%) |
|  | *Marital status* | |  |  |  |  |  |  |  |  |  |  |  |  |  |  |  |  |  |  |  |  |  |  |  |  |  |  |
|  |  | Married | 4,621 | (87.3%) |  | 5,017 | (89.2%) |  | 4,578 | (86.1%) |  | 5,185 | (86.0%) |  | 5,305 | (84.0%) |  | 5,408 | (85.4%) |  | 5,450 | (84.7%) |  | 5,568 | (84.1%) |  | 41,132 | (85.7%) |
|  |  | Non-married | 659 | (12.4%) |  | 608 | (10.8%) |  | 738 | (13.9%) |  | 846 | (14.0%) |  | 1,014 | (16.0%) |  | 928 | (14.6%) |  | 985 | (15.3%) |  | 1,055 | (15.9%) |  | 6,833 | (14.2%) |
|  |  | Missing | 14 | (0.3%) |  | 0 | (0.0%) |  | 1 | (0.0%) |  | 0 | (0.0%) |  | 0 | (0.0%) |  | 0 | (0.0%) |  | 0 | (0.0%) |  | 0 | (0.0%) |  | 15 | (0.0%) |
|  | *Household size* | |  |  |  |  |  |  |  |  |  |  |  |  |  |  |  |  |  |  |  |  |  |  |  |  |  |  |
|  |  | 1 | 400 | (7.6%) |  | 331 | (5.9%) |  | 528 | (9.9%) |  | 449 | (7.4%) |  | 571 | (9.0%) |  | 534 | (8.4%) |  | 517 | (8.0%) |  | 535 | (8.1%) |  | 3,865 | (8.1%) |
|  |  | 2 | 866 | (16.4%) |  | 892 | (15.9%) |  | 1,129 | (21.2%) |  | 1,193 | (19.8%) |  | 1,301 | (20.6%) |  | 1,403 | (22.1%) |  | 1,390 | (21.6%) |  | 1,557 | (23.5%) |  | 9,731 | (20.3%) |
|  |  | 3 | 1,095 | (20.7%) |  | 1,210 | (21.5%) |  | 1,255 | (23.6%) |  | 1,609 | (26.7%) |  | 1,672 | (26.5%) |  | 1,699 | (26.8%) |  | 1,778 | (27.6%) |  | 1,847 | (27.9%) |  | 12,165 | (25.4%) |
|  |  | 4 | 1,533 | (29.0%) |  | 1,841 | (32.7%) |  | 1,414 | (26.6%) |  | 1,717 | (28.5%) |  | 1,776 | (28.1%) |  | 1,777 | (28.0%) |  | 1,819 | (28.3%) |  | 1,763 | (26.6%) |  | 13,640 | (28.4%) |
|  |  | 5 or above | 1,400 | (26.4%) |  | 1,351 | (24.0%) |  | 991 | (18.6%) |  | 1,063 | (17.6%) |  | 999 | (15.8%) |  | 923 | (14.6%) |  | 931 | (14.5%) |  | 921 | (13.9%) |  | 8,579 | (17.9%) |
|  | *Education* | |  |  |  |  |  |  |  |  |  |  |  |  |  |  |  |  |  |  |  |  |  |  |  |  |  |  |
|  |  | Below primary level | 694 | (13.1%) |  | 617 | (11.0%) |  | 708 | (13.3%) |  | 576 | (9.6%) |  | 462 | (7.3%) |  | 385 | (6.1%) |  | 382 | (5.9%) |  | 342 | (5.2%) |  | 4,166 | (8.7%) |
|  |  | Primary level | 2,070 | (39.1%) |  | 2,218 | (39.4%) |  | 1,955 | (36.8%) |  | 1,988 | (33.0%) |  | 2,094 | (33.1%) |  | 1,993 | (31.5%) |  | 1,864 | (29.0%) |  | 1,887 | (28.5%) |  | 16,069 | (33.5%) |
|  |  | Secondary level | 2,010 | (38.0%) |  | 2,271 | (40.4%) |  | 2,107 | (39.6%) |  | 2,764 | (45.8%) |  | 3,083 | (48.8%) |  | 3,236 | (51.1%) |  | 3,325 | (51.7%) |  | 3,461 | (52.3%) |  | 22,257 | (46.4%) |
|  |  | Tertiary level | 508 | (9.6%) |  | 519 | (9.2%) |  | 542 | (10.2%) |  | 703 | (11.7%) |  | 680 | (10.8%) |  | 722 | (11.4%) |  | 864 | (13.4%) |  | 933 | (14.1%) |  | 5,471 | (11.4%) |
|  |  | Missing | 12 | (0.2%) |  | 0 | (0.0%) |  | 5 | (0.1%) |  | 0 | (0.0%) |  | 0 | (0.0%) |  | 0 | (0.0%) |  | 0 | (0.0%) |  | 0 | (0.0%) |  | 17 | (0.0%) |
|  | *Household income (HKD)* | |  |  |  |  |  |  |  |  |  |  |  |  |  |  |  |  |  |  |  |  |  |  |  |  |  |  |
|  |  | $9999 or less | 1,455 | (27.5%) |  | 1,540 | (27.4%) |  | 1,696 | (31.9%) |  | 1,422 | (23.6%) |  | 1,728 | (27.3%) |  | 1,796 | (28.3%) |  | 1,410 | (21.9%) |  | 1,231 | (18.6%) |  | 12,278 | (25.6%) |
|  |  | $10000-24999 | 1,988 | (37.6%) |  | 2,145 | (38.1%) |  | 1,919 | (36.1%) |  | 2,520 | (41.8%) |  | 2,462 | (39.0%) |  | 2,498 | (39.4%) |  | 2,348 | (36.5%) |  | 2,233 | (33.7%) |  | 18,113 | (37.8%) |
|  |  | $25000-49999 | 1,144 | (21.6%) |  | 1,382 | (24.6%) |  | 1,001 | (18.8%) |  | 1,522 | (25.2%) |  | 1,495 | (23.7%) |  | 1,433 | (22.6%) |  | 1,938 | (30.1%) |  | 2,152 | (32.5%) |  | 12,067 | (25.2%) |
|  |  | $50000 or above | 444 | (8.4%) |  | 558 | (9.9%) |  | 347 | (6.5%) |  | 425 | (7.0%) |  | 634 | (10.0%) |  | 609 | (9.6%) |  | 739 | (11.5%) |  | 1,007 | (15.2%) |  | 4,763 | (9.9%) |
|  |  | Missing | 263 | (5.0%) |  | 0 | (0.0%) |  | 354 | (6.7%) |  | 142 | (2.4%) |  | 0 | (0.0%) |  | 0 | (0.0%) |  | 0 | (0.0%) |  | 0 | (0.0%) |  | 759 | (1.6%) |
|  | *Diabetes* | |  |  |  |  |  |  |  |  |  |  |  |  |  |  |  |  |  |  |  |  |  |  |  |  |  |  |
|  |  | No | 5,015 | (94.7%) |  | 5,270 | (93.7%) |  | 4,974 | (93.5%) |  | 5,628 | (93.3%) |  | 5,747 | (90.9%) |  | 5,732 | (90.5%) |  | 5,758 | (89.5%) |  | 5,901 | (89.1%) |  | 44,025 | (91.8%) |
|  |  | Yes | 279 | (5.3%) |  | 355 | (6.3%) |  | 343 | (6.5%) |  | 403 | (6.7%) |  | 572 | (9.1%) |  | 604 | (9.5%) |  | 677 | (10.5%) |  | 722 | (10.9%) |  | 3,955 | (8.2%) |
