# Supplementary material for: Gender-specific trends of educational inequality in diagnosed diabetes from 1999 to 2014 in Hong Kong: a serial cross-sectional study of 97,481 community-dwelling Chinese adults
Source: Popul Health Metr. 2021 Oct 10;19:37. doi: 10.1186/s12963-021-00268-x (PMC8504033; doi:10.1186/s12963-021-00268-x)
Supplement: Supplementary file 4 — Additional file 4. Relative and absolute educational inequalities in diabetes across years. RII and SII measures across 8 survey years. [file 12963_2021_268_MOESM4_ESM.docx]

| **Additional file 4.** **Relative and absolute educational inequalities in diabetes across years** | | | | | | |  | |  | | |  | | |  | | |  | | |  |
| --- | --- | --- | --- | --- | --- | --- | --- | --- | --- | --- | --- | --- | --- | --- | --- | --- | --- | --- | --- | --- | --- |
|  |  |  | 1999 | 2001 | 2002 | 2005 | | 2008 | | | 2009 | | | 2011 | | | 2014 | | | Annual change ^c^ | |
| All | |  |  |  |  |  | |  | | |  | | |  | | |  | | |  | |
|  | RII (95% CI) ^a^ | | 1.38 (1.00-1.91)* | 1.02 (0.78-1.34) | 1.42 (1.06-1.90)* | | 1.61 (1.23-2.12)*** | | | 1.48 (1.17-1.86)*** | | | 1.67 (1.34-2.09)*** | | | 1.64 (1.35-2.00)*** | | | 1.59 (1.30-1.94)*** | | 1.02 (1.00-1.03)* |
|  | SII (95% CI) ^a^ | | 2.26 (0.36-4.17)* | 0.68 (-1.32-2.68) | 3.10 (1.06-5.15)** | | 4.49 (2.56-6.41)*** | | | 4.21 (2.18-6.24)*** | | | 5.44 (3.37-7.50)*** | | | 5.57 (3.53-7.61)*** | | | 5.43 (3.50-7.35)*** | | 0.19 (0.05-0.33)** |
| Female | | |  |  |  | |  | | |  | | |  | | |  | | |  | |  |
|  | RII (95% CI) ^b^ | | 1.56 (0.99-2.46) | 1.38 (0.94-2.02) | 1.83 (1.21-2.75)** | | 1.87 (1.24-2.81)** | | | 2.23 (1.57-3.19)*** | | | 2.29 (1.62-3.24)*** | | | 2.44 (1.83-3.24)*** | | | 1.80 (1.33-2.42)*** | | 1.04 (1.02-1.07)*** |
|  | SII (95% CI) ^b^ | | 2.99 (0.15-5.83)* | 3.25 (0.32-6.18)* | 5.29 (2.44-8.14)*** | | 4.99 (2.07-7.90)*** | | | 7.39 (4.61-10.18)*** | | | 7.63 (4.73-10.53)*** | | | 9.21 (6.47-11.96)*** | | | 6.26 (3.73-8.79)*** | | 0.36 (0.16-0.56)*** |
| Male | | |  |  |  | |  | | |  | | |  | | |  | | |  | |  |
|  | RII (95% CI) ^b^ | | 1.30 (0.83-2.03) | 0.74 (0.51-1.08) | 1.04 (0.69-1.55) | | 1.41 (0.98-2.02) | | | 1.02 (0.76-1.39) | | | 1.25 (0.93-1.67) | | | 1.09 (0.83-1.43) | | | 1.40 (1.07-1.82)* | | 1.01 (0.99-1.04) |
|  | SII (95% CI) ^b^ | | 1.55 (-0.94-4.05) | -1.94 (-4.60-0.72) | 0.19 (-2.59-2.96) | | 2.54 (0.03-5.06)* | | | 0.55 (-2.42-3.52) | | | 2.26 (-0.67-5.19) | | | 1.19 (-1.85-4.23) | | | 3.56 (0.65-6.47)* | | 0.17 (-0.03-0.37) |
| ^a^ Age group, gender, marital status, household size, education fractional rank score, and household income fractional rank score were included | | | | | | | | | | | | | | | | | | | | | |
| ^b^ Age group, marital status, household size, education fractional rank score, and household income fractional rank score were included | | | | | | | | | | | | | | | | | | | | | |
| ^c^ A two-way interaction between education fractional rank score and survey years was further included for assessing annual RII change; while a three-way interaction among age groups, education fractional rank score, and survey years was further included for assessing annual SII change | | | | | | | | | | | | | | | | | | | | | |
