# Supplementary material for: Gender-specific trends of educational inequality in diagnosed diabetes from 1999 to 2014 in Hong Kong: a serial cross-sectional study of 97,481 community-dwelling Chinese adults
Source: Popul Health Metr. 2021 Oct 10;19:37. doi: 10.1186/s12963-021-00268-x (PMC8504033; doi:10.1186/s12963-021-00268-x)
Supplement: Supplementary file 5 — Additional file 5. Relative and absolute educational inequalities in diabetes across years after further adjustments for household income. RII and SII measures across 8 survey years after adjustment for household income level. [file 12963_2021_268_MOESM5_ESM.docx]

| **Additional file 5.** **Relative and absolute educational inequalities in diabetes across years after further adjustments for household income** | | | | | | |  | |  | | |  | |  |
| --- | --- | --- | --- | --- | --- | --- | --- | --- | --- | --- | --- | --- | --- | --- |
|  |  | 1999 | 2001 | 2002 | 2005 | 2008 | | 2009 | | 2011 | 2014 | | Annual change ^c^ | |
| All | |  |  |  |  |  | |  | |  |  | |  | |
|  | RII (95% CI) ^a^ | 1.42 (1.00-2.01)* | 1.03 (0.79-1.36) | 1.51 (1.11-2.06)** | 1.63 (1.22-2.16)*** | 1.37 (1.09-1.74)** | | 1.65 (1.31-2.08)*** | | 1.68 (1.37-2.06)*** | 1.61 (1.31-1.98)*** | | 1.01 (0.98-1.05) | |
|  | SII (95% CI) ^a^ | 1.35 (-0.66-3.35) | 0.49 (-1.57-2.55) | 3.21 (1.02-5.39)** | 4.39 (2.41-6.38)*** | 3.13 (1.08-5.18)** | | 5.15 (3.03-7.27)*** | | 5.57 (3.52-7.63)*** | 5.08 (3.11-7.04)*** | | 0.13 (-0.15-0.42) | |
| Female | |  |  |  |  |  | |  | |  |  | |  | |
|  | RII (95% CI) ^b^ | 1.47 (0.90-2.38) | 1.39 (0.95-2.05) | 1.94 (1.26-3.01)** | 1.92 (1.26-2.94)** | 2.13 (1.49-3.04)*** | | 2.22 (1.56-3.16)*** | | 2.44 (1.82-3.26)*** | 1.82 (1.34-2.48)*** | | 1.08 (1.03-1.13)** | |
|  | SII (95% CI) ^b^ | 1.95 (-1.07-4.96) | 2.70 (-0.25-5.66) | 5.65 (2.48-8.82)*** | 5.32 (2.37-8.26)*** | 6.81 (4.07-9.56)*** | | 7.50 (4.61-10.40)*** | | 9.79 (7.01-12.56)*** | 5.98 (3.37-8.59)*** | | 0.49 (0.08-0.90)* | |
| Male | |  |  |  |  |  | |  | |  |  | |  | |
|  | RII (95% CI) ^b^ | 1.34 (0.83-2.15) | 0.74 (0.50-1.08) | 1.13 (0.73-1.73) | 1.39 (0.95-2.04) | 0.92 (0.67-1.27) | | 1.25 (0.92-1.69) | | 1.14 (0.86-1.51) | 1.41 (1.07-1.85)* | | 0.99 (0.94-1.04) | |
|  | SII (95% CI) ^b^ | 1.12 (-1.50-3.73) | -1.21 (-3.88-1.47) | 0.14 (-3.01-3.30) | 2.01 (-0.65-4.68) | -0.47 (-3.54-2.60) | | 1.87 (-1.07-4.81) | | 1.18 (-1.96-4.32) | 3.16 (0.18-6.14)* | | -0.01 (-0.40-0.39) | |
| ^a^ Age group, gender, marital status, household size, education fractional rank score, and household income fractional rank score were included | | | | | | | | | | | | | | |
| ^b^ Age group, marital status, household size, education fractional rank score, and household income fractional rank score were included | | | | | | | | | | | | | | |
| ^c^ Two two-way interactions, one between education fractional rank score and survey years and another one between household income fractional rank score and survey years, were further included for assessing annual RII change; while two three-way interactions, one among age groups, education fractional rank score, and survey years and another one among age groups, household income fractional rank score, and survey years, were further included for assessing annual SII change | | | | | | | | | | | | | | |
|  | | | | | | | | | | | | | | |
